# Behavioral and emotional difficulties and HIV treatment outcomes among HIV-infected children in rural southwestern China

**DOI:** 10.1186/s13034-023-00601-2

**Published:** 2023-04-18

**Authors:** Yesheng Zhou, Kailing Tang, Hongyan Lu, Hongli Chen, Haomin Xie, Zeyu Li, Jinghua Huang, Ningye Fang, Siya Chen, Hong Wang, Qin He, Huanhuan Chen, Xiu Liu, Guanghua Lan, Qiuying Zhu, Yi Chen, Xiangjun Zhang, Yuhua Ruan, Shujia Liang

**Affiliations:** 1 State Key Laboratory of Infectious Disease Prevention and Control (SKLID), National Center for AIDS/STD Control and Prevention (NCAIDS), Chinese Center for Disease Control and Prevention (China CDC), Collaborative Innovation Center for Diagnosis and Treatment of Infectious Diseases, Beijing, China; 2 Guangxi Key Laboratory of Major Infectious Disease Prevention Control and Biosafety Emergency Response, Guangxi Center for Disease Control and Prevention, Nanning, 530028 China; 3 Department of Clinical Pharmacy and Translational Science, College of Pharmacy, University of Tennessee Health Science Center, 881 Madison Avenue, Memphis, TN 38163 USA

**Keywords:** Behavioral and emotional problems, Strengths and Difficulties Questionnaire, Children, HIV, Antiretroviral therapy, Treatment outcome

## Abstract

**Background:**

Previous studies have not clearly demonstrated the impact of behavioral and emotional problems (BEDs) on treatment outcomes among HIV-infected children on antiretroviral therapy (ART). This study aimed to describe the prevalence of BEDs among this population and identify the factors associated with HIV treatment outcomes.

**Methods:**

This cross-sectional study was conducted in Guangxi, China, between July and August 2021. HIV-infected children answered questionnaires about BEDs, physical health, social support, and whether they have missed doses in the past month. BEDs were assessed using the Chinese version of the self-reported Strengths and Difficulties Questionnaire (SDQ-C). The self-reported survey data were linked to participants’ HIV care information that was obtained from the national surveillance database. Univariate and multivariate logistic regression models were used to identify factors that were associated with missed doses in the past month and virological failure.

**Results:**

The study sample was 325 HIV-infected children. HIV-infected children had a higher proportion of abnormal scores on SDQ-C total difficulties compared to their peers in the general population (16.9 vs 10.0%; *P* = 0.002). An abnormal SDQ-C total difficulties score (AOR = 2.06, 95%CI: 1.10–3.88) and infrequency of receiving assistance and support from parents over the past 3 months (AOR = 1.85, 95%CI: 1.12–3.06) were significantly associated with missed doses in the past month. Between the ages of 14–17 years (AOR = 2.66, 95% CI: 1.37–5.16), female (AOR = 2.21, 95% CI: 1.20–4.08), and suboptimal adherence (AOR = 2.45, 95% CI: 1.32–4.57) were significantly associated with virological failure.

**Conclusions:**

Children’s mental health plays a role in HIV treatment outcomes. Psychological interventions should be promoted in pediatric HIV care clinics to improve children’s mental health status and HIV treatment outcomes.

## Background

In 2015, WHO recommended initiating antiretroviral therapy (ART) for all children with HIV immediately after the diagnosis regardless of their clinical stages and CD4 cell counts [1]. Although enormous efforts have been taken to promote early ART worldwide, ART coverage among HIV-infected children remained low. Among an estimated 1.7 million HIV-infected children aged 15 years and younger in 2021, only 52.0% of them received ART [2]. In China, ART coverage among HIV-infected children was relatively higher as a result of the effective expansion of HIV treatment in recent years, for example, by the end of 2020, 95.2% of 7,935 HIV-infected children younger than 15 years old were on ART [3]. In general, pediatric HIV treatment faced unique challenges. The viral suppression rate decreased markedly after ART initiation from 64% in the first year and 62% in the second year to 59% in the third year [4]. Being unable to maintain viral suppression often indicates virological failure. Adherence, a major factor associated with viral suppression [5], was reported particularly low with a range of 53% to 84% among HIV-infected adolescents [6]. Further, the risk of loss to follow-up was higher among adolescents aged 11 to 19 years compared to adults and young children [7].

There are three groups regarding children affected by HIV in the literature, including HIV-exposed but uninfected children (HEU), HIV-infected children who acquired HIV through the mother-to-child transmission route, and HIV-infected children who acquired HIV through the sexual transmission route. Children from the three groups may differ in their neuropsychiatric presentations, specifically attentional, cognitive, and emotional dysregulation [8–10]. Compared to HIV-unexposed, uninfected children (HUU), both HEU children and HIV-infected children face mental health challenges [11, 12]. Behavioral and emotional difficulties (BEDs) refer to a variety of behavioral and emotional abnormalities that occur in children before the age of 18 and predominantly manifest as anxiety, fear, depression, hyperactivity, impulsivity, and disobedience [13]. HEU children had an increased risk of having psychiatric disorders compared to HUU children [12]. Further, previous studies reported that HIV-infected children were more likely to suffer from BEDs than their HIV-negative peers [14, 15]. The reasons included the physical and neurological effects of HIV infection on behavior, emotion, and cognition as well as social and emotional effects due to various stressors HIV-infected children often face, which were associated with HIV infection and treatment [15, 16]. Some social challenges faced by HIV-infected children included poverty, HIV stigma, relationship difficulty related to HIV disclosure, and the school system’s inability to meet their needs [17]. In addition, previous studies reported that parental involvement, parents’ perception and attitude toward medication taking were associated with children’s medication adherence [18, 19].

However, the literature has not clearly demonstrated the effects of BEDs on treatment outcomes in HIV-infected children who were receiving ART or reported inconsistent results [14, 20–23]. Two previous studies reported that BEDs were associated with nonadherence among HIV-infected adolescents [20, 21]. Another study found that HIV-infected adolescents who screened positive for depression, post-traumatic stress disorder, and substance use had a higher likelihood of an unsuppressed viral load [22]. Other previous studies reported inconsistent results, for example, BEDs were only associated with adherence in HIV-infected male children [23], and no association between BEDs and virological failure [14]. In addition, previous studies related to BEDs of HIV-infected children have been mainly conducted in Africa and Western countries [11, 24]. A gap existed in the HIV-infected children BEDs literature in Asian studies. This study aimed to describe the prevalence of BEDs among HIV-infected children on ART in rural southwestern China and identify the factors associated with HIV treatment outcomes, specifically, medication adherence and virological failure.

## Materials and methods

### Study design and participants

The study was conducted in Guangxi Zhuang Autonomous Region in southwestern China (Guangxi). Guangxi, which borders Vietnam and is close to the “Golden Triangle”, is impacted disproportionately by injection drug use historically [25]. HIV transmission in Guangxi was mainly through injection drug use between 1996 and 2005 [25]. After years of efforts in the promotion of HIV testing, prevention, and treatment, HIV reported cases showed a decreased trend from 2013 to 2017 [25]. However, reported HIV cases are still high among all provinces and autonomous regions in China in recent years [25]. Therefore, mother-to-child cases are comparably high due to the high HIV disease burden in adults. This cross-sectional study was conducted between July and August 2021. HIV-infected children were recruited from the pediatric HIV clinic of the Guangxi Center for Disease Control and Prevention (Guangxi CDC). Children who met the following eligibility criteria were recruited for this study: (1) HIV positive; (2) being 11 and 17 years old at the study recruitment; (3) having initiated ART for at least 6 months; and (4) being willing to participant in the study and providing written informed consent from themselves and their parents or guardians. This study did not include HIV-infected children who were admitted to an inpatient care or with serious physical injury.

### Data collection

#### Baseline characteristics

Trained HIV clinic staff provided guidance to participants who completed a 10-min questionnaire for data collection. The questionnaire consisted of three parts: BEDs, physical health, and social support. Upon completion of the questionnaire, blood sample was collected by a nurse for viral load and CD4 cell count testing. Transportation compensation of 30 RMB (approximately $5 USD) was provided to each participant. The clinic staff informed all participants and their parents or guardians the blood test results when the results became available.

All HIV positive cases were required to report through a national surveillance portal in China, the National Free Antiretroviral Treatment Program (NFATP) database. We linked questionnaire data to NFATP database to collect the following information: sociodemographic data (age, sex, and route of HIV infection), HIV clinical characteristics (WHO clinical stage before ART), laboratory data (CD4 cell count before ART), HIV care information (age at ART initiation, year of ART initiation, initial ART regimen, and time on ART).

#### Behavioral and emotional difficulties (BEDs)

BEDs were assessed using the Chinese version of the self-reported Strengths and Difficulties Questionnaire (SDQ-C), which was publicly available on the official website of SDQ (http://www.sdqinfo.com). This tool has been validated in China and other countries and has been used in HIV-infected children in previous studies [24, 27]. The SDQ-C contains 25 items, which were categorized into five subscales: emotional symptoms, conduct problems, hyperactivity/inattention, peer problems, and prosocial behavior. The severity level of each item was rated on a 3-point scale (0 = not true, 1 = somewhat true, and 2 = certainly true). A questionnaire with one or more unanswered questions was considered invalid, therefore, were not included in the analysis. The total difficulties score was calculated by summing up the four subscale scores (emotional symptoms, conduct problems, hyperactivity/inattention, and peer problems). In general, a higher score represents greater difficulties for the total difficulty score and the four subscales. While for the prosocial subscale, a lower score indicates greater difficulties. We also calculated Cronbach’s alpha to measure the internal consistency of subscales. In our sample, Cronbach’s alpha scores for the total difficulties was 0.75 and were 0.50 and 0.72, respectively for emotional symptoms and hyperactivity/inattention subscales. The Cronbach’s alpha was comparably low for conduct problems (a = 0.41) and peer problems (a = 0.11) subscales. In addition, the proportions of HIV-infected children in the ‘normal’, ‘borderline’, and ‘abnormal’ categories were calculated respectively using the norms for Chinese children (Shanghai norms) [28].

#### Physical health

The Chinese version of the Medical Outcomes Study HIV Health Survey (MOS-HIV), which was translated by Yu et al. [29], showed good reliability and validity in Chinese people living with HIV [30]. In this study, physical health was measured using the sections of the MOS-HIV on physical function and general health perceptions. The scores of all domains were obtained by an established calculation method and then transformed into a 0–100 scale [31]. A higher score represents better physical functioning and well-being. For multi-item scales, mean substitution was generally used for missing items if the missing items were ≤ 50%. In our sample, Cronbach’s alpha scores were good for the physical functioning score (a = 0.81) and the general health score (a = 0.57).

#### Social support

The questionnaire collected the information of social support participants have received. Participants reported the frequency of having received assistance and support in the past 3 months from parents and other various sources including siblings, relatives, neighbors, friends, medical and nursing staff/CDC staff, teachers, fellow students, net friends, and other HIV-infected patients. The frequency of receiving support from each source was rated on a 5-point scale (1 = never, 2 = occasionally, 3 = sometimes, 4 = often, and 5 = always).

#### HIV treatment outcomes

ART medication adherence and virological indicators were the treatment outcomes. Adherence was measured by children’s answers on a question about whether they had missed doses in the past month. Virological failure was defined as HIV-1 RNA viral load of ≥ 50 copies/ml using the blood test result for this study [32].

### Laboratory tests

All viral load and CD4 cell count tests were conducted in the Guangxi CDC laboratory. Viral load was tested using the COBAS TaqMan 96 system (Roche Molecular Systems, Branchburg, NJ, USA) and CD4 cell count was assessed using the Alere PimaTM Analyzer (Abbott Laboratories, Germany).

### Statistical analysis

All paper-based survey data were entered into EpiData software version 3.1 (The EpiData Association, Odense, Denmark) and two research staff cross-checked for errors. Statistical analyses were conducted using SAS V9.4 (SAS Institute Inc., Cary, NC, USA). The internal consistency of the questionnaire was assessed using Cronbach’s α coefficient. We reported the means and standard deviations (SD) for continuous variables. Comparisons were conducted using independent T-tests for continuous variables and Chi-square tests for categorical variables. Univariate and multivariate logistic regression models were used to investigate potential factors that were associated with missed doses in the past month and virological failure. Further, we constructed a multivariate logistic regression model stepwise to select variables independently associated with missed doses in the past month and virological failure. All tests were two-tailed, and a *P*-value of < 0.05 was considered statistically significant.

## Results

### Demographic and HIV care characteristics

Among all questionnaires that were collected from 331 participants, 325 (98.2%) responses were used for analysis with the rest had at least one missing item. Table 1 demonstrated participants’ demographic and HIV care information at baseline. The mean age of participants at the time of data collection was 14.4 years (SD = 1.9). Males accounted for 52.0% of the sample. Most participants were infected through mother-to-child transmission (96.0%). Further, 70.2% of participants were at the WHO clinical stages I/II and 68.9% of participants had a CD4 cell count of ≥ 200 cells/mm^3^ at the ART initiation. Approximately half of participants (52.9%) started ART at younger than 5 years old, and 55.7% of participants started ART in 2012 or after. A total of 65.2% of participants were on the first-line ART regimens at the time of data collection. More than half of participants (59.1%) had less than 10 years of ART treatment.

**Table 1:** Demographic and HIV care characteristics of HIV-infected children on ART in southwestern China

Variables	Number	%
Total	325	100.0
Age at study survey (years)		
11–13	142	43.7
14–17	183	56.3
Sex		
Male	169	52.0
Female	156	48.0
Route of HIV infection		
Mother-to-child transmission	312	96.0
Others	13	4.0
WHO clinical stage before ART		
I/II	228	70.2
III/IV	97	29.9
CD4 cell count before ART (cells/mm^3^)		
≥ 200	224	68.9
< 200	101	31.1
Age at ART initiation (years)		
< 5	172	52.9
≥ 5	153	47.1
Year of ART initiation		
< 2012	144	44.3
≥ 2012	181	55.7
Initial ART regimen		
The first-line ART	212	65.2
The second-line ART	113	34.8
Time on ART (years)		
< 10	192	59.1
≥ 10	133	40.9

*WHO*  World Health Organization, *ART*  Antiretroviral therapy.

### SDQ-C scores

Overall, 16.9% (55/325) of participants scored in the abnormal range on the total difficulties scale. For subscales, 12.9% (42/325), 11.4% (37/325), 6.2% (20/325), 7.4% (24/325), and 15.4% (50/325) of participants were in the abnormal ranges of emotional symptoms, conduct problems, hyperactivity/inattention, peer problems, and prosocial behavior subscales, respectively.

Compared to Shanghai norms of the SDQ-C for children in the general population, this study sample demonstrated a higher proportion of abnormal scores on total difficulties (16.9 vs 10.0%; *P* = 0.002), emotional symptoms (12.9 vs 7.1%; *P* = 0.003), conduct problems (11.4 vs 7.4%; *P* = 0.035) and prosocial behavior (15.4 vs 7.8%; *P* < 0.001, Table 2).

**Table 2:** Proportions of participants with SDQ-C abnormal scores compared to the Shanghai norms

	Normal range	SDQ-C Abnormal Score (%, n/N)	
		This study	Shanghai norms
Total difficulties**	0–14	16.9 (55/325)	10.0 (69/690)
Emotional symptoms**	0–4	12.9 (42/325)	7.1 (49/690)
Conduct problems*	0–3	11.4 (37/325)	7.4 (51/690)
Hyperactivity/Inattention	0–5	6.2 (20/325)	7.4 (51/690)
Peer problems	0–4	7.4 (24/325)	6.0 (41/690)
Prosocial behavior**	6–10	15.4 (50/325)	7.8 (54/690)

*SDQ-C* Chinese version of the self-reported Strengths and Difficulties Questionnaire

Chi-square tests were performed for all comparisons. * *P* < 0.05, ** *P* < 0.01

### Gender differences in SDQ-C mean scores

We compared male and female participants on their scores of total difficulties and subscales. Female participants showed significantly higher scores than males on emotional symptoms (3.08 ± 2.45 vs 2.41 ± 2.19, *P* = 0.010) and prosocial behavior (7.12 ± 2.13 vs 6.24 ± 2.05, *P* < 0.001), while male participants exhibited significantly higher scores on peer problems (3.58 ± 1.56 vs 3.13 ± 1.44, *P* = 0.007, Table 3).

**Table 3:** SDQ-C mean scores between males and females of HIV-infected children in southwestern China

	Scores (Mean, SD)		*t*	*P*-value
	Male	Female		
Total difficulties score	12.26 (5.15)	12.17 (5.61)	0.15	0.880
Emotional symptoms*	2.41 (2.19)	3.08 (2.45)	−2.60	0.010
Conduct problems	2.67 (1.66)	2.39 (1.69)	1.51	0.133
Hyperactivity/Inattention	3.60 (1.83)	3.57 (1.93)	0.14	0.886
Peer problems**	3.58 (1.56)	3.13 (1.44)	2.70	0.007
Prosocial behavior**	6.24 (2.05)	7.12 (2.13)	−3.79	0.000

*SDQ-C* Chinese version of the self-reported Strengths and Difficulties Questionnaire, *SD* Standard deviation

Independent sample T-tests were performed for all comparisons. *P < 0.05, **P < 0.01

### Factors associated with missed doses in the past month

A total of 90 out of 325 participants reported they had missed doses in the past month (27.7%). Results of the multivariate logistic regression showed that an abnormal total difficulties score (AOR: 2.06, 95% CI: 1.10–3.88) and infrequency of receiving assistance and support from parents over the past 3 months (AOR: 1.85, 95% CI: 1.12–3.06) were significantly associated with missed doses in the past month (Table 4).

**Table 4:** Factors associated with missed doses in the past month among HIV-infected children on ART in southwestern China

Variables	N	Missed doses in the past month, N (%)	OR (95% CI)	*P*-value	AOR (95% CI)	*P*-value
Total	325	90 (27.7)				
Age at study survey (years)						
11–13	142	36 (25.4)	1.00			
14–17	183	54 (29.5)	1.23 (0.75–2.02)	0.407		
Sex						
Female	156	38 (24.4)	1.00			
Male	169	52 (30.8)	1.38 (0.85–2.25)	0.198		
Route of HIV infection						
Mother-to-child transmission	312	88 (28.2)	1.00			
Others	13	2 (15.4)	0.46 (0.10–2.13)	0.323		
WHO clinical stage before ART						
I/II	228	60 (26.3)	1.00			
III/IV	97	30 (30.9)	1.25 (0.74–2.11)	0.396		
CD4 cell count before ART (cells/mm^3^)						
≥ 200	224	58 (25.9)	1.00			
< 200	101	32 (31.7)	1.33 (0.79–2.22)	0.281		
Age at ART initiation (years)						
< 5	172	45 (26.2)	1.00			
≥ 5	153	45 (29.4)	1.18 (0.72–1.91)	0.514		
Year of ART initiation						
< 2012	144	35 (24.3)	1.00			
≥ 2012	181	55 (30.4)	1.36 (0.83–2.23)	0.224		
Initial ART regimen						
The first-line ART	212	65 (30.7)	1.00			
The second-line ART	113	25 (22.1)	0.64 (0.38–1.09)	0.103		
Time on ART (years)						
< 10	192	58 (30.2)	1.00			
≥ 10	133	32 (24.1)	0.73 (0.44–1.21)	0.224		
Total difficulties score						
Normal	225	52 (23.1)	1.00		1.00	
Borderline	45	16 (35.6)	1.84 (0.93–3.64)	0.082	1.82 (0.91–3.63)	0.091
Abnormal	55	22 (40.0)	2.22 (1.19–4.13)	0.012	2.06 (1.10–3.88)	0.024
Physical functioning score						
≥ 80	229	56 (24.5)	1.00			
< 80	96	34 (35.4)	1.69 (1.01–2.84)	0.045		
General health score						
≥ 80	166	40 (24.1)	1.00			
< 80	159	50 (31.4)	1.45 (0.89–2.36)	0.140		
Frequency of having received assistance and support from parents over the past 3 months						
Often or always	161	34 (21.1)	1.00		1.00	
Never, occasionally or sometimes	164	56 (34.1)	1.94 (1.18–3.18)	0.009	1.85 (1.12–3.06)	0.017
Frequency of having received assistance and support from other sources over the past 3 months						
Often or always	245	70 (28.6)	1.00			
Never, occasionally, or sometimes	80	20 (25.3)	0.83 (0.47–1.48)	0.536		

*OR* Odds ratio, *AOR* Adjusted odds ratio, *CI* Confidence interval, *WHO* World Health Organization, *ART* Antiretroviral therapy

### Factors associated with virological failure

A total of 56 out of 325 HIV-infected children experienced virological failure (17.2%). Results of the multivariate logistic regression showed that age of between 14 and 17 years old (AOR: 2.66, 95% CI: 1.37–5.16), being female (AOR: 2.21, 95% CI: 1.20–4.08), and having missed doses in the past month (AOR: 2.45, 95% CI: 1.32–4.57) were significantly associated with virological failure (Table 5).

**Table 5:** Factors associated with virological failure among HIV-infected children on ART in southwestern China

Variables	N	Viral load ≥ 50 copies/mL, N (%)	OR (95% CI)	*P*-value	AOR (95% CI)	*P*-value
Total	325	56 (17.2)				
Age at study survey (years)						
11–13	142	14 (9.9)	1.00		1.00	
14–17	183	42 (23.0)	2.72 (1.42–5.22)	0.003	2.66 (1.37–5.16)	0.004
Sex						
Male	169	21 (12.4)	1.00		1.00	
Female	156	35 (22.4)	2.04 (1.13–3.69)	0.018	2.21 (1.20–4.08)	0.012
Route of HIV infection						
Mother-to-child transmission	312	55 (17.6)	1.00			
Others	13	1 (7.7)	0.39 (0.05–3.06)	0.370		
WHO clinical stage before ART						
I/II	228	38 (16.7)	1.00			
III/IV	97	18 (18.6)	1.14 (0.61–2.12)	0.680		
CD4 cell count before ART (cells/mm^3^)						
≥ 200	224	40 (17.9)	1.00			
< 200	101	16 (15.8)	0.87 (0.46–1.63)	0.656		
Age at ART initiation (years)						
< 5	172	25 (14.5)	1.00			
≥ 5	153	31 (20.3)	1.49 (0.84–2.67)	0.174		
Year of ART initiation						
< 2012	144	25 (17.4)	1.00			
≥ 2012	181	31 (17.1)	0.98 (0.55–1.76)	0.956		
Initial ART regimen						
The first-line ART	212	42 (19.8)	1.00			
The second-line ART	113	14 (12.4)	0.57 (0.30–1.10)	0.094		
Time on ART (years)						
< 10	192	31 (16.1)	1.00			
≥ 10	133	25 (18.8)	1.20 (0.67–2.15)	0.534		
Total difficulties score						
Normal	225	35 (15.6)	1.00			
Borderline	45	8 (17.8)	1.17 (0.50–2.73)	0.710		
Abnormal	55	13 (23.6)	1.68 (0.82–3.45)	0.157		
Physical functioning score						
≥ 80	229	40 (17.5)	1.00			
< 80	96	16 (16.7)	0.95 (0.50–1.79)	0.862		
General health score						
≥ 80	166	27 (16.3)	1.00			
< 80	159	29 (18.2)	1.15 (0.65–2.04)	0.638		
Frequency of having received assistance and support from parents over the past 3 months						
Often or always	161	25 (15.5)	1.00			
Never, occasionally or sometimes	164	31 (18.9)	1.27 (0.71–2.26)	0.421		
Frequency of having received assistance and support from other sources over the past 3 months						
Often or always	245	46 (18.8)	1.00			
Never, occasionally or sometimes	80	10 (12.7)	0.62 (0.30–1.29)	0.200		
Missed doses in the past month						
No	235	32 (13.6)	1.00		1.00	
Yes	90	24 (26.7)	2.31 (1.27–4.19)	0.006	2.45 (1.32–4.57)	0.005

*OR* odds ratio, *AOR* adjusted odds ratio, *CI* confidence interval, *WHO* World Health Organization, *ART* antiretroviral therapy

## Discussion

The prevalence of 16.9% for an abnormal score of the total difficulties scale was consistent with previous studies conducted among HIV-infected children on ART, which had a range between 6.4% to 43.6% [14, 20, 33, 34]. The study that generated Shanghai norms used a representative sample of children aged 11–17 years from 12 of the 19 districts in Shanghai [28]. HIV-infected children on ART have shown higher prevalence of BEDs compared to Shanghai norms [28]. Further, compared to Shanghai norms, HIV-infected children on ART in our study were more likely to develop emotional symptoms, had higher rates of conduct problems, and were less likely to have prosocial behavior [28]. This study did not find a significant difference between HIV-infected children and Shanghai norms on peer problems and hyperactivity [28]. Future studies could explore in depth the reasons that result in the differences in BEDs between HIV-infected children and children in the general population.

This study found a gender difference in three BEDs subscales. Females showed a higher score on emotional symptoms, while males demonstrated more peer problems and poor prosocial behavior. These results were all consistent with the findings in the general population aged 12–18 years in the previous studies [35]. In previous studies investigating HIV-infected children, one study reported males were more likely to suffer from emotional symptoms [23], and no gender differences in peer problems were found in a study conducted in Africa [15]. The gender difference in BEDs for HIV-infected children has implications in pediatric HIV care, in which care providers must pay attention to the unique needs of male and female children and offer specific trainings and coping skills to children with BEDs.

This study found that HIV-infected children with abnormal SDQ-C total difficulties score were more likely missing doses for ART medications in the past month. This result was consistent with previous findings demonstrating that BEDs were negatively associated with medication adherence [20, 21, 36]. Pediatric HIV care providers must work with adolescents and their parents or guardians to develop a plan to maintain a good medication adherence and identify any possible obstacles for adherence at ART initiation. For children who could not adhere to their medication due to BEDs, psychological professionals must be referred to these patients. Further, this study also found that parents’ support was critical for HIV-infected children to adhere to their medications. This result was consistent with previous findings [37]. Parents played a vital role in supporting HIV-infected children financially, physically, and emotionally, also most children relied on their parents to access HIV care and to manage their medications [38]. Therefore, pediatric HIV care providers must work together with parents in addressing HIV-related issues that children encounter, for example, provide trainings to patients on medication management.

Some factors were found to be associated with virological failure, including age of 14–17, being female, and having missed doses in the past month. Some previous studies also found that older children had a higher risk of virological failure [39, 40], which could be explained by the poorer adherence and higher likelihood of drug resistance in older children [39, 41]. Future studies are still needed to further testify this association and verify the reasons between age and virological failure because other previous studies did not detect this association [42, 43]. In this study, females were 2.21 times more likely to experience virological failure compared to males. This result was consistent with previous studies, which showed that females were roughly 2.50 times more likely to experience virological failure than males [44, 45]. However, other previous studies reported either a higher risk of virological failure in males [43], or no association between gender and virological failure [39, 42]. The inconclusive result on this association needs to be further assessed in large and diverse samples in future studies. Further, the association between nonadherence and virological failure has been well proven in previous studies [39, 41, 42].

Although this study did not identify a direct association between BEDs and virological failure, children’s behavioral and emotional status were undoubtedly a critical factor on HIV outcomes because the indirect association through adherence was identified. Further, children’s behavioral and emotional status was a vital factor involving with their daily life and well-being. HIV-infected children were vulnerable population for BEDs, pediatric HIV care providers and parents must pay more attention to children’s social, behavioral, and emotional needs and provide support in their daily lives, not only on HIV care and medication management, but also on coping with social, emotional, and relationship issues.

This study has limitations. The cross-sectional study design was unable to generate causal relationships between BEDs and HIV treatment outcomes. Future studies could consider examining the associations using experimental or longitudinal study designs. This study sample was HIV-infected children aged 11 to 19 years resided in Guangxi. The study findings could not be generalized to other populations, such as younger children with HIV and HEU children. This study did not include social and environmental factors that potentially could also have an impact on children’s behaviors and HIV treatment outcomes. Future studies could explore these factors in relation to HIV-infected children’s behaviors and health status. In addition, this study did not investigate HEU children. Future studies could explore the impact of HIV exposure on children’s health by specifying the three groups because HEU children are also a vulnerable group that may need attention for research and psychological interventions. Further, considering participants' capability of providing accurate information, we measured medication adherence using a survey question asking about missed doses in the past month. However, this self-reported method was susceptible to recall bias, also, this adherence measure might not fully reflect participants’ adherence status. Although MOS-HIV has not been validated in HIV-infected children, it has shown good reliability and validity in adults living with HIV/AIDS [30]. In addition, the internal consistency of the self-reported conduct problems and peer problems subscales in this study was relatively low, which was consistent with previous studies that were conducted among Chinese children and in other cultures [46, 47]. It could be explained by the small number of items for each domain; however, these items may represent more heterogeneous constructs than we intended to measure. Despite the low internal consistency of the two subscales, this scale has been widely applied in many cultures with different languages. In this sample, mother-to-child transmission is the major transmission route. Families and parents of our participants may encounter unique challenges relative to children’s care and medication utilization due to some other factors, such as parents’ HIV status, mental health status, general health status, poverty, stigma, death, etc. All these factors may have an impact on children’s health and HIV care. Therefore, in the survey for support sources, various sources of receiving support were included. Future studies that investigate HIV treatment outcomes, such as medication adherence may consider the potential impact of parental factors. Further, future studies could also consider investigating parental responses and views on children’s behaviors and emotional symptoms as complementary information to children’s self-reports.

## Conclusions

HIV-infected children’s behavioral and emotional status was a crucial factor and plays a role in the HIV treatment and care. Psychological screening tests must be provided in pediatric HIV care clinics to detect children with behavioral and emotional disorders. Moreover, psychological interventions should be promoted in pediatric HIV care clinics to both HIV-infected children and their families with the goals to improve children’s mental health status and HIV treatment outcomes.

## Data Availability

Not applicable.

## Abbreviations

BEDs: Behavioral and emotional problems
ART: Antiretroviral therapy
Guangxi CDC: Guangxi Center for Disease Control and Prevention
SDQ-C: Chinese version of the self-reported Strengths and Difficulties Questionnaire
WHO: World Health Organization
SD: Standard deviation
OR: Odds ratio
AOR: Adjusted odds ratio
CI: Confidence interval
